# Artesunate repurposing for cytomegalovirus management: a systematic review of efficacy, mechanisms, and perspectives

**DOI:** 10.3389/fcimb.2026.1815679

**Published:** 2026-05-07

**Authors:** Haohao Zhang, Fanchao Zhou, Jianping Song, Changsheng Deng, Xiao He, Suiqin Ni

**Affiliations:** 1 Department of Clinical Pharmacy, Guangzhou First People’s Hospital, Guangzhou Medical University, Guangzhou, China; 2 Artemisinin Research Center, Guangzhou University of Chinese Medicine, Guangzhou, China

**Keywords:** antiviral, artemisinin, artesunate, CMV infection, cytomegalovirus, hcmv

## Abstract

**Background:**

Cytomegalovirus (CMV) is a prevalent opportunistic pathogen associated with significant morbidity and mortality in immunocompromised individuals, particularly transplant recipients and neonates. Current antiviral therapies are often constrained by substantial toxicity and the emergence of drug resistance. Artesunate (ART), a proven antimalarial agent with established safety profiles, has been explored for potential repurposing against CMV infection, though its anti-CMV pharmacodynamics remain incompletely elucidated.

**Methods:**

This systematic review synthesized evidence from 46 studies published through December 2025, covering *in vitro*, *in vivo*, and clinical studies, to evaluate the efficacy, mechanisms of action, and translational challenges of ART-based anti-CMV strategies.

**Results:**

ART exhibits potent anti-CMV activity *in vitro*, with no reported cross-resistance to conventional antiviral agents. Structure–activity relationship analysis indicates that the endoperoxide bridge and hemisuccinate side chain may contribute to its antiviral effects. While optimized ART derivatives show improved potency, they remain in the preclinical research stage. Clinical evidence for ART is limited, primarily derived from small-scale and combination therapy studies, with inconsistent outcomes typically marked by an initial reduction in viral load rather than sustained complete clearance. Mechanistically, ART may exert anti-CMV effects through multiple host-regulated pathways, including NF-κB inhibition, G0/G1 cell cycle arrest, and modulation of mitochondrial dynamics.

**Conclusions:**

The short plasma half-life of ART may represent a pharmacokinetic limitation to its clinical application. Further research, including formulation optimization, targeted pharmacokinetic/pharmacodynamic studies, and well-controlled clinical trials, is warranted to evaluate the potential role of ART in CMV management.

## 1 Introduction

### 1.1 Epidemiology of CMV

Human cytomegalovirus (HCMV, often abbreviated as CMV), also known as human herpesvirus 5 (HHV-5), is a double-stranded DNA β-herpesvirus belonging to the family *Herpesviridae*. CMV infection is highly prevalent worldwide, with seroprevalence rates ranging from 60% to almost 100% in adult populations, varying from geographic location and socioeconomic conditions (Zuhair et al., 2019). In immunocompetent individuals, primary CMV infection is typically asymptomatic or mild, and the virus establishes a lifelong latent state characterized by minimal viral shedding due to its immune evasion mechanisms (Schwartz and Stern-Ginossar, 2023). In contrast, immunocompromised patients such as HIV-infected persons, recipients of solid-organ transplants or hematopoietic stem cell transplants (HSCT), are at high risk of severe and potentially fatal CMV disease. In these groups, viral reactivation or primary infection often begins with viremia and can progress to serious end-organ complications, including gastroenteritis, hepatitis, nephritis, retinitis, pneumonia, and neurological inflammation, which significantly contribute to patient mortality (Boeckh and Geballe, 2011).

Furthermore, CMV is also a leading infectious cause of congenital disease, with an estimated birth prevalence between 0.3% and 1.2% (Fowler et al., 2022). Approximately 10% to 20% of congenitally infected neonates exhibit clinical manifestations at birth, such as microcephaly, hepatosplenomegaly, and pneumonia. Of these symptomatic infants, about 30% to 40% die from neonatal complications (Jenks et al., 2021). Notably, 15% to 25% of infants who are initially asymptomatic or mildly symptomatic develop long-term sequelae, most commonly sensorineural hearing loss or neurodevelopmental delay (Sapuan et al., 2022). Collectively, CMV infections pose a substantial health burden, particularly for the immunocompromised population.

### 1.2 Virology of CMV

**Figure 1:**
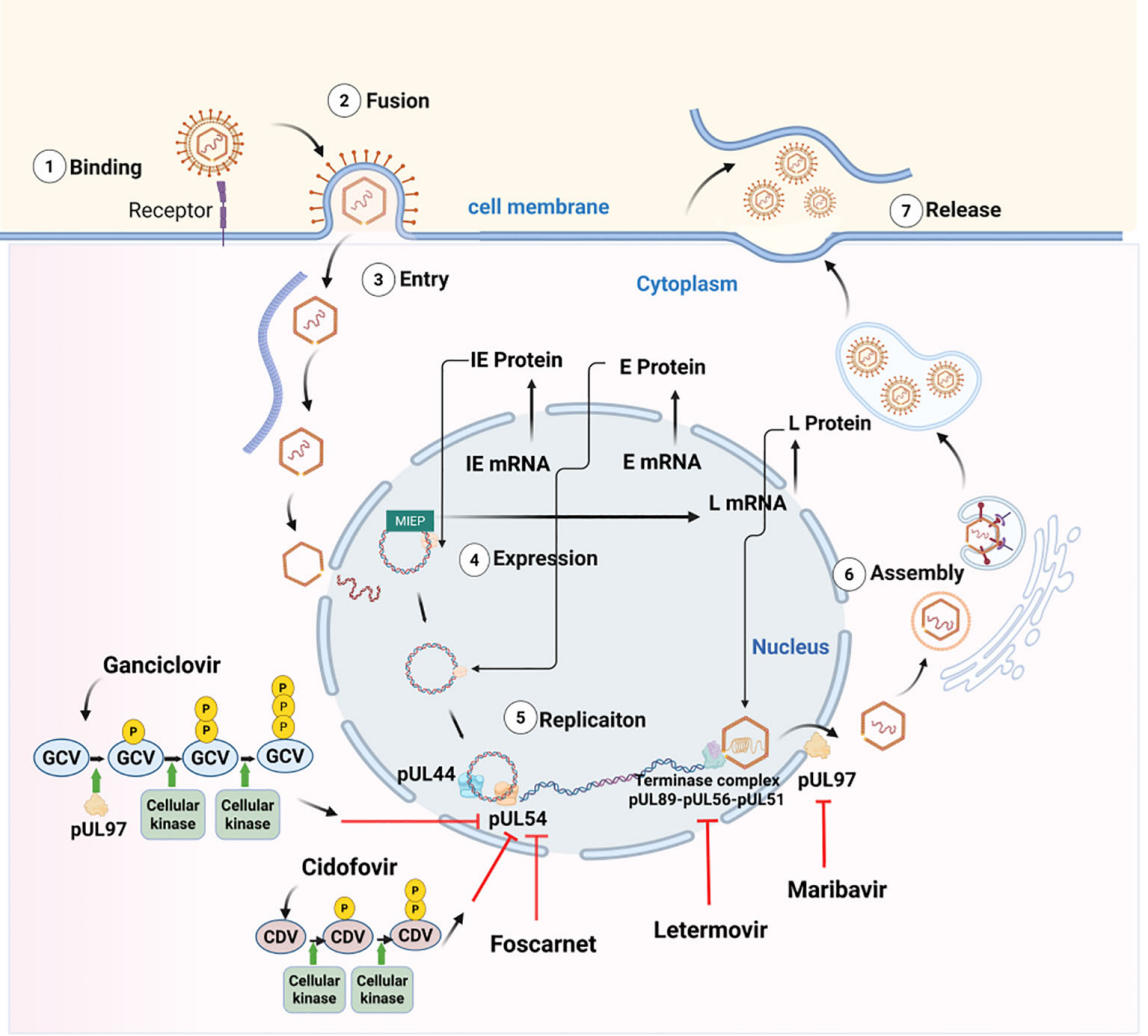
The CMV replication cycle and mechanisms of conventional antiviral agents. Created with BioRender.com.

The CMV replication cycle proceeds through highly coordinated and sequential stages. Virion entry is mediated by glycoprotein complexes on the virion surface, primarily glycoprotein B (gB) and the gH–gL complex, which interact with host receptors (e.g., EGFR, integrins, PDGFRα) to facilitate attachment and membrane fusion (Tyl et al., 2022; Viswanathan et al., 2011). Following entry, the virion particle exploits the host microtubule network for intracellular trafficking and nuclear delivery (Ogawa-Goto et al., 2003). Transcription initiates at the major immediate−early promoter (MIEP), driven by host RNA polymerases. Activation of the MIEP acts as a key molecular switch governing the transition from viral latency to lytic replication (Stinski and Isomura, 2008). After MIEP activation, CMV genes are expressed in a temporal cascade, including immediate−early (IE), early (E), and late (L) genes (Hou et al., 2016). Genome replication follows a single-copy mechanism. Newly assembled nucleocapsids acquire tegument proteins in the cytoplasm and undergo final envelopment in Golgi−derived membranes, where viral glycoproteins are processed. Mature virions are subsequently released extracellularly through membrane fusion, enabling continued infection of neighboring cells (Griffiths et al., 2015). The entire replication cycle of CMV is relatively slow, typically taking more than 24 hours, with an additional 24–36 hours often required to achieve high viral titers (Salvant et al., 1998; Ye et al., 2020) (**Figure 1**).

### 1.3 Current status of CMV therapeutics

To date, no licensed vaccine for CMV is available, underscoring the critical role of antiviral therapy in CMV management. Ganciclovir (GCV) remains the first-line anti-CMV agent. Following cellular uptake, GCV is initially phosphorylated by the viral kinase pUL97 and subsequently by host cellular kinases to its active triphosphate form. This metabolite competitively inhibits viral DNA polymerase pUL54, thereby suppressing viral DNA synthesis. The prodrug valganciclovir (VGCV) offers markedly improved oral bioavailability compared to oral GCV and shares the same mechanism of action. These agents, alongside conventional drugs such as foscarnet (FOS) and cidofovir (CDV), all primarily target the viral DNA polymerase pUL54 (Chen et al., 2022). However, their clinical use is severely constrained by significant toxicities, including hematologic suppression and nephrotoxicity. These adverse events often force dose adjustment or therapy discontinuation, which leads to treatment failure (Haidar et al., 2020). Two novel antiviral agents have been approved for transplant recipients. Letermovir (LTV), an inhibitor of the viral terminase complex of pUL89, pUL56, and pUL51, has demonstrated efficacy for CMV prophylaxis in allogeneic HSCT recipients. Nevertheless, evidence supporting its use in active infection remains insufficient (Marty et al., 2017). Maribavir (MBV), which targets the viral kinase pUL97, is indicated for refractory or resistant CMV infections in solid organ transplant or HSCT recipients (Kang, 2022) (**Figure 1**). Immunotherapeutic strategies, such as adoptive transfer of CMV-specific T cells, have shown promise as adjunctive therapy in HSCT patients, but are constrained by high costs, time-consuming preparation, and complex logistics (Valencia Deray et al., 2024).

Despite these therapeutic advances, the management of CMV remains a considerable challenge due to the increasing emergence of antiviral resistance, driven primarily by mutations in the UL97 and UL54 genes, particularly in transplant recipients (Chou et al., 2018). Moreover, treatment options are especially scarce for specific populations such as pregnant women and neonates (Boucoiran et al., 2021). Consequently, there persists​ an urgent need to develop novel anti-CMV agents that may pose a higher barrier to resistance and offer broader applicability, including in special populations.

### 1.4 Artesunate and its pharmacokinetic profile

Artemisinin is a sesquiterpene lactone compound extracted from *Artemisia annua L*, characterized by a 2,4-trioxane moiety with a distinctive peroxide bridge, a structural feature critical for its bioactivity (van Agtmael et al., 1999). The landmark discovery of artemisinin by Tu Youyou in the 1970s established it as the global gold standard for malaria treatment, owing to its potent therapeutic profile with IC50 of anti-malaria down to nanomoler (Tu, 2016). Among artemisinin derivatives, artesunate (ART) is one of the most widely used in intravenous antimalarial treatment and has been extensively studied. Its chemical structure includes a hemisuccinate group, which enhances stability, aqueous solubility, and bioavailability compared to the parent compound (Newton et al., 2000). ART can be administered via intravenous (IV), intramuscular (IM), oral, and rectal routes, allowing flexible clinical use. Oral bioavailability of ART is approximately 80%, much higher than that of 19% for artemisinin (Gautam et al., 2009). Following administration, both artemisinin and ART are rapidly hydrolyzed by plasma esterases to dihydroartemisinin (DHA), the major active metabolite. DHA exhibits a plasma protein binding rate of 88–93% (Xie et al., 2009), undergoes further metabolism via hepatic glucuronidation, and is mainly excreted in urine (Morris et al., 2011). While DHA accounts for most of the anti-malarial activity, ART itself may contribute more significantly following IV administration (Davis et al., 2001). After IV injection at the routine adult dose of 120 mg, ART reaches an extremely high peak plasma concentration (Cmax) of 35.6 μM, but is rapidly eliminated with an elimination half-life (T1/2) of 2.2 min (Batty et al., 1998). DHA appears in plasma within 5 minutes, with a time to peak concentration (Tmax) of 0.5–15 minutes, Cmax of 5.3–10.6 μM, and T1/2 of 21–64 minutes. IM injection of ART yields slower drug release, with a Tmax of 12 minutes and T1/2 of 41 minutes. Oral ART typically achieves a Tmax of 1.5 hours and T1/2 of 43 minutes (Medhi et al., 2009; Byakika-Kibwika et al., 2012; Awad et al., 2004). Overall, ART exhibits a very short half-life regardless of the administration route.

### 1.5 Safety profile of ART

The well-established safety profile of artemisinin-based drugs is supported by decades of global use in malaria control. Adverse events are primarily mild, transient, and dose-dependent gastrointestinal, hematologic, or neurologic reactions (Efferth and Kaina, 2010; Zou et al., 2020). Nevertheless, preclinical studies raised concerns over potential teratogenicity during early organogenesis (White et al., 2006). The World Health Organization (WHO) recommends artemisinin-based combination therapies (ACTs) for uncomplicated malaria only during the second and third trimesters of pregnancy. For severe malaria, intravenous artesunate (2.4–3 mg/kg/dose) remains the first-line treatment for all patient groups, including pregnant women in any trimester, prioritizing maternal survival (WHO, 2022). A recent meta−analysis aggregated 12 cohorts totaling 34,178 pregnancies, of which 737 had confirmed first−trimester exposure to ACTs, reported a lower incidence of adverse outcomes in the exposed versus non-exposed group (5.7% vs. 8.9%; aHR 0.71, 95%CI 0.49–1.03), with no evidence of embryotoxicity or teratogenicity (Saito et al., 2023). These data further support the safety of artemisinin derivatives even in early pregnancy, while continued pharmacovigilance across diverse populations remains warranted.

### 1.6 Potential of ART in CMV therapy

Beyond their established antimalarial use, artemisinin derivatives exhibit broad pharmacological activities, including anticancer (Chaturvedi et al., 2010; Lu and Efferth, 2021), antiviral (Efferth, 2018; Chemaly et al., 2019), and anti-inflammatory effects (Gao et al., 2024; Zhou et al., 2025). They show notable inhibition against double-stranded DNA herpesviruses such as CMV, Epstein-Barr virus (EBV), and human herpesvirus 6A (HHV-6). Among them, ART exhibits potent anti-CMV activity, positioning it as a promising antiviral candidate. Owing to the compelling *in vitro* antiviral performance, ART has been explored for treating drug-resistant or refractory CMV infections, especially in transplant recipients who are at high risk of life-threatening CMV disease. Current guidelines recommend ART as a second-line or salvage therapy for drug-resistant CMV in HSCT patients (Ljungman et al., 2019; Chinese Society of Clinical Oncology, 2025). However, existing research on the efficiency of ART against CMV remains fragmented. Its precise mechanism of action and pharmacodynamic properties are not yet fully understood. This review systematically summarizes the therapeutic potential, pharmacodynamic characteristics, and proposed mechanisms of ART against CMV.

## 2 Methods

This review follows the PRISMA 2020 checklist (Page et al., 2021). Literature searches and screenings, as well as bias assessments, were performed independently by two researchers. In case of disagreement, a third researcher was responsible for making the final judgment. Studies were included if they met the following PICOS criteria.

1. Population: CMV infection
2. Intervention: Use of artemisinin-based compounds
3. Comparison: Negative/blank control or standard antiviral agents (GCV, FOS, etc.) as positive control.
4. Outcomes: Antiviral efficacy, including half maximal inhibitory concentration (IC50), half maximal effective concentration (EC50), half maximal cytotoxic concentration (CC50), selectivity Index (SI= CC50/EC50), synergistic effects with standard antiviral drugs, viral load inhibition, viral clearance rate, virological response, and clinical outcome.
5. Studies: All experimental and clinical studies, including *in vitro* experiments, *in vivo* animal studies, clinical case reports, case series, cohort studies, and clinical trials.

The exclusion criteria were defined as follows: (1) duplicate records, (2) review articles, conference abstracts, letters, or expert opinions, (3) studies irrelevant to the anti-CMV activity of artemisinin compounds, (4) studies with insufficient data, including intervention information details or incomplete/untraceable core outcome data.

### 2.1 Search strategy

Literature published up to December 2025 was searched in PubMed and Web of Science. Keywords included (‘artemisinin’ OR ‘artesunate’ OR ‘dihydroartemisinin’ OR ‘artemether’ OR ‘arteether’) AND (‘cytomegalovirus’ OR ‘CMV’ OR ‘HCMV’). Additional relevant studies have been manually supplemented based on the cited references.

### 2.2 Data extraction

Information was sorted out according to basic study information, model characteristics, intervention details, core antiviral efficacy outcomes, and proposed mechanism of action. Key information and findings were synthesized and visualized.

### 2.3 Risk of bias assessment

The risk of bias of included studies was assessed using study type-specific tools: the QUIN risk of bias tool for *in vitro* studies (Sheth et al., 2024), SYRCLE’s risk of bias tool for *in vivo* animal studies (Hooijmans et al., 2014), and the JBI Critical Appraisal Checklists for clinical studies (Stern et al., 2020).

## 3 Results

**Figure 2:**
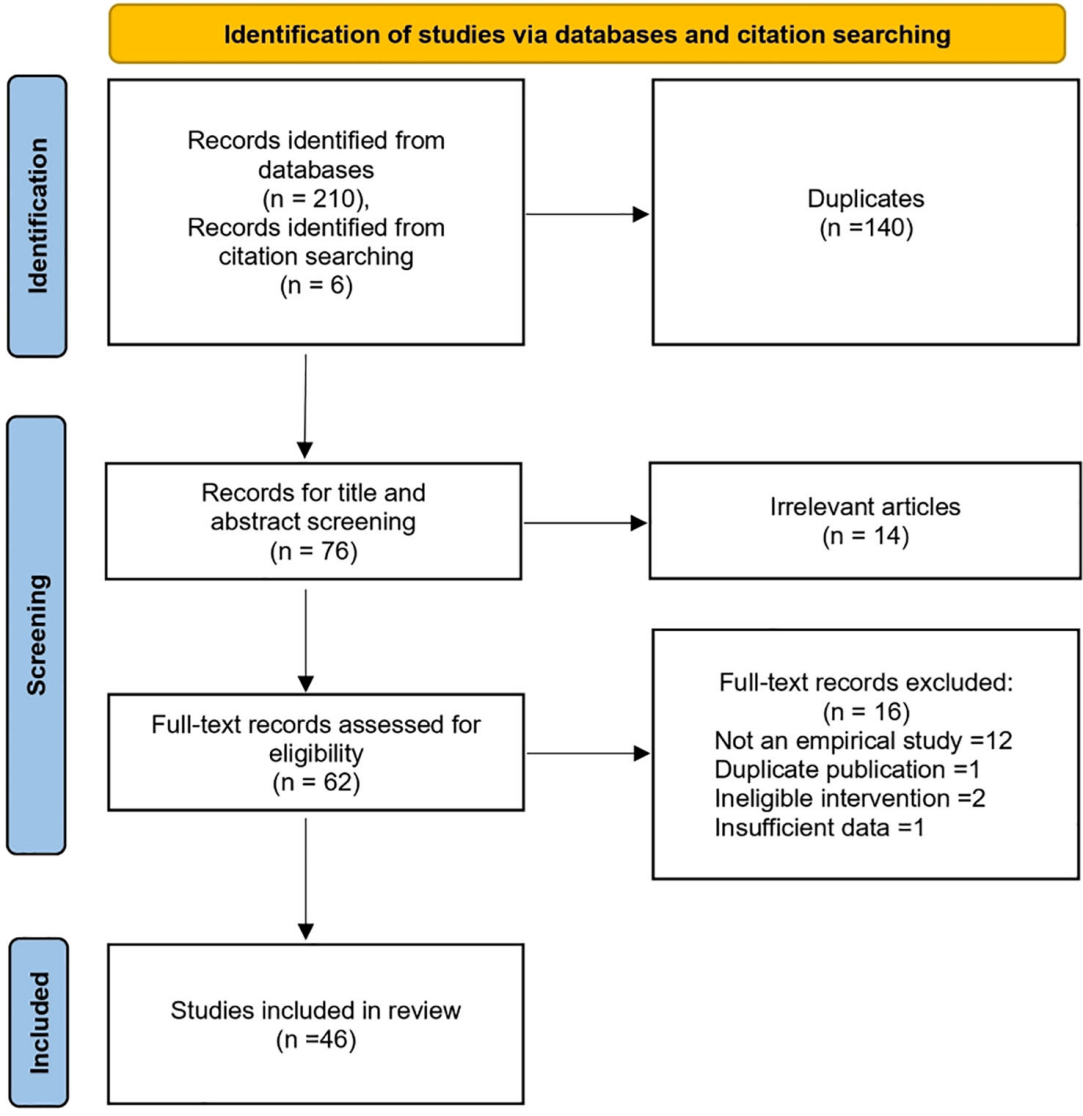
PRISMA flow diagram of the literature search and study selection.

**Figure 3:**
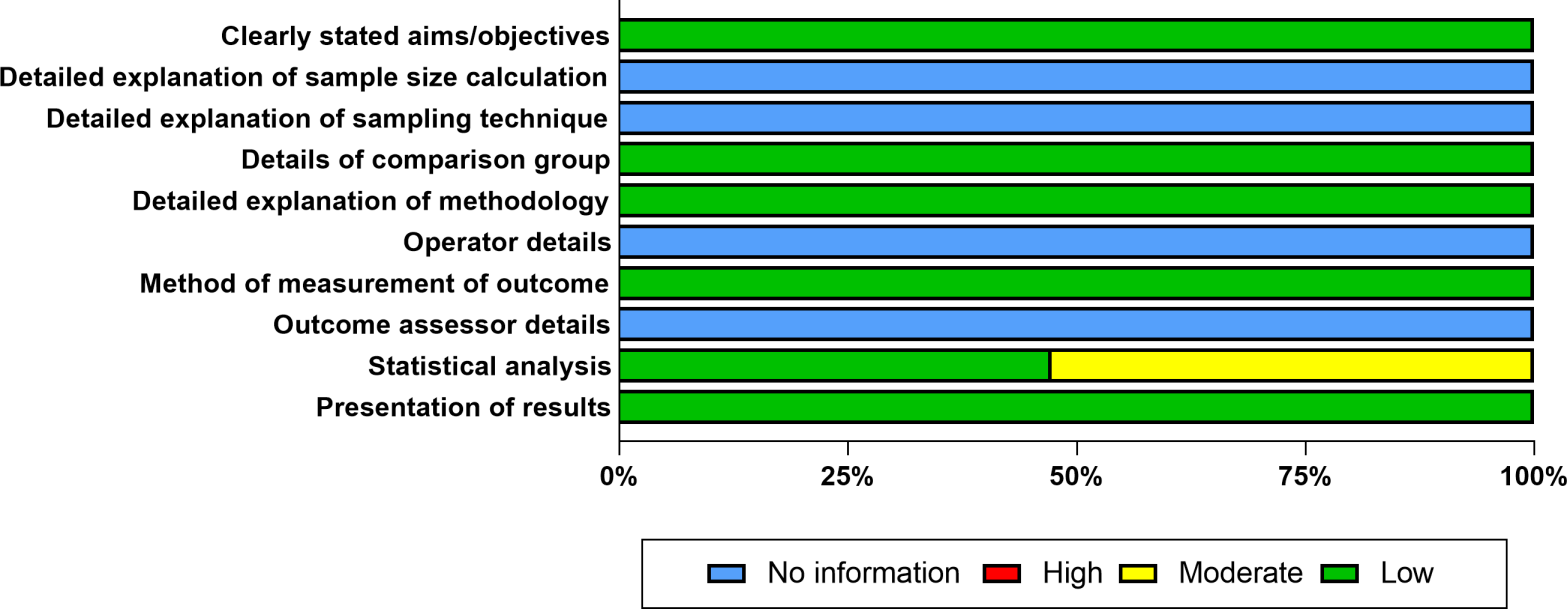
Summary bar plot for the risk of bias assessment of *in vitro* studies.

**Figure 4:**
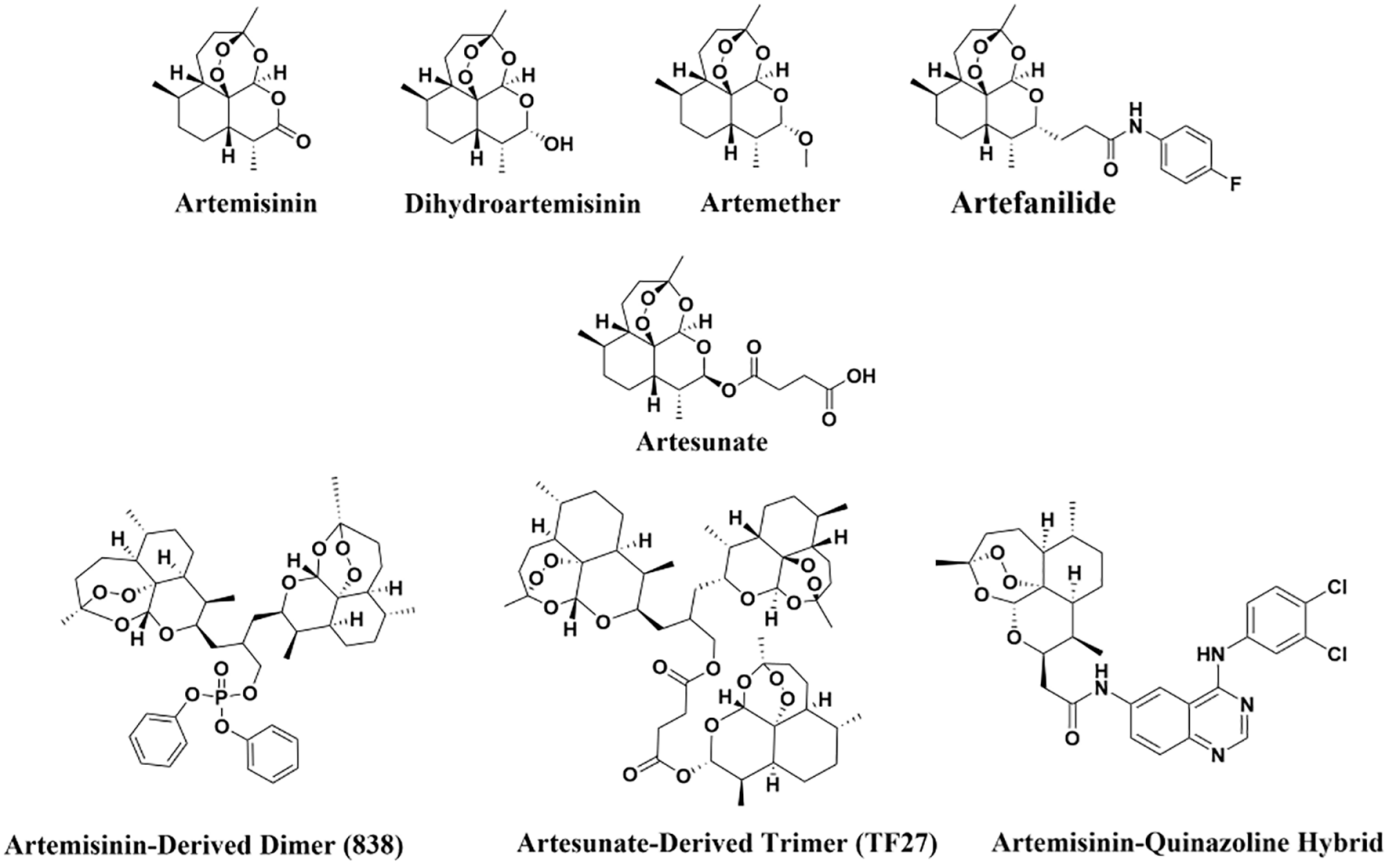
Representative artemisinin-based compounds screened for anti-CMV activity.

After screening and deduplication, a total of 46 preclinical and clinical studies were included, among which 4 studies involved both *in vitro* and *in vivo* experiments (**Figure 2**). Risk of bias assessments for these studies are presented in **Figure 3** and the **Supplementary Materials**. A range of artemisinin derivatives has been evaluated for their anti-CMV activity, with representative compounds including monomers (artemisinin, ART, DHA, artemether, and artefanilide), dimer and trimer derivatives (compound 838 and TF27), and hybrid molecules (artemisinin-quinazoline hybrid) (**Figure 4****).**

### 3.1 *In vitro* study

Risk of bias assessment using the QUIN tool indicated that the 36 included *in vitro* studies presented an overall low risk of bias across most core methodological domains and outcome measurement procedures. The major high-risk issue was insufficient reporting of statistical analysis methods (**Figure 3**).

*In vitro* antiviral activity is a key criterion for identifying candidate therapeutics. Although CMV can infect a diverse range of human cell types *in vivo*, it replicates most efficiently in human fibroblasts and endothelial cells under *in vitro* conditions. As congenital CMV infection is a major clinical concern, *ex vivo* placental models are also frequently used to evaluate novel antiviral agents. The primary pharmacodynamic properties of artemisinin compounds against CMV reported in these studies were summarized (**Table 1**).

**Table 1:** *In vitro* studies on ART compounds against CMV.

Study	Cell model	Virus strain	Intervention and antiviral efficacy	Findings and mechanism
(Efferth et al., 2002)	HELF, HFF	AD169, Towne	ART (IC50 5.8 ± 0.4 μM for GCV-sensitive strain, IC50 6.9 ± 0.2 μM for GCV-resistant strain)	Inhibited CMV at the early stage; suppressed NF-κB, Sp1, Akt, and p70S6K signaling.
(Arav-Boger et al., 2010)	HELF, HFF	Towne, clinical isolate	artemisinin (EC50 16.8 ± 4.0 μM, CC50 72.4 ± 15.7 μM, SI 4.3 ± 1.4), ART (EC50 18.5 ± 5.2 μM, CC50 77.5 ± 14.4 μM, SI 4.2 ± 2.2), artemether (EC50 5.3 ± 2.7 μM, CC 50 18.4 ± 7.5 μM, SI 3.5 ± 2.2), artefanilide (EC50 8.1 ± 2.2 μM, CC50 44.9 ± 3.4 μM, SI 5.5 ± 1.6), dimer sulfone carbamate (EC50 0.06 ± 0.00 μM, CC50 28.1 ± 9.6 μM, SI 508 ± 173), dimer primary alcohol (EC50 0.15 ± 0.02 μM, CC50 57 ± 2.3 μM, SI 380 ± 53), GCV (EC50 5.6 ± 0.2 μM, CC50 247 ± 33.4 μM, SI 44 ± 6.2)	Inhibited IE1 gene expression; exerted antiviral effects at the early phase of CMV infection
(Flobinus et al., 2014)	MRC-5	AD169, Clinical isolate	ART (IC50 22.19 ± 9.89 μM in 0% FCS, IC50 3.46 ± 1.14 μM in 10% FCS), DHA (IC50 47.06 ± 13.79 μM in 0% FCS, IC50 16.51 ± 7.29 μM in 10% FCS), artemisinin showed no activity	Antiviral activity was reduced in serum-starved and iron-depleted culture conditions; fractional dosing enhanced the antiviral efficacy of ART and DHA
(He et al., 2012)	HFF	Towne	ART (EC50 6.6 ± 0.4 μM), dimer compound 838 (EC50 0.04 ± 0.003 μM)	Inhibited virus replication during the first 24h; reduced viral yield and IE gene expression; ART was a reversible inhibitor, while compound 838 was irreversible. ART and 838 were synergistic with GCV.
(Hahn et al., 2020)	HFF	AD169	artemisinin-derived monomeric BG95 (EC50 0.10 ± 0.00 μM), AC98 (EC50 0.58 ± 0.27 μM) and AC173 (EC50 1.93 ± 0.85 μM), trimer compound TF27 (EC50 0.04 ± 0.00 μM), all of compounds CC50 >100 μM	Downregulated mitochondrial protein expression, potential targets included mitochondrial proteins (HSP60, PHB, ATP5A1, MDH2, and MTCH2), NF-κB (p105 submit, p65), and UL132, etc.
(Roy et al., 2015)	HFF; HCT116	Towne	ART (EC50 12.2 μM), dimer compound 606 (EC50 nanomolar range)	Induced G1 cell cycle arrest, reduced CDK2/4/6 expression, and pRb phosphorylation.
(Jacquet et al., 2020)	MRC-5; ARPE-19; *ex vivo* placenta model	AD169, TB40/E, VHL/E, clinical isolate	ART (*In vitro*, EC50 1.3–3.5 μM; in 1st trimester placenta, EC50 4.33–11.51 μM) trimer compound TF27 (*In vitro*, EC50 0.01–0.32 μM; in 1st trimester placenta, EC50 0.020–0.28 μM)	Antiviral potency of ART and TF27 was consistent across all tested strains. TF27 showed greater efficacy in the 1st trimester placenta than in the 3rd trimester placenta.
(Held et al., 2017)	HFF	AD169	artesunic acid-quinazoline hybrids (EC50 down to 0.1 ± 0.0 μM)	Fluorescence of hybrids for molecular imaging; greater antiviral potency than artesunic acid and GCV.
(Herrmann et al., 2023)	HFF	AD169	artemisinin–benzimidazole hybrids (EC50 down to 0.07 ± 0.00 μM)	Induced mitochondrial structural changes; the endoperoxide bridge was important for the bioactivity.

HFF, primary human foreskin fibroblasts; HELF, human embryonic lung fibroblasts; HCT116, human colorectal carcinoma cell line; ARPE-19, human adult retinal pigment epithelial cell line; MRC-5, human embryonic lung fibroblast cell line.

#### 3.1.1 Activities of different artemisinin compounds

While monomeric artemisinin compounds, including ART, DHA, and artemisinin, share a common core, their anti−CMV potency varies markedly. ART exhibits consistently potent anti−CMV activity *in vitro*, with IC50 values ranging from 1.30 to 22.19 μM under various experimental conditions, a potency comparable to that of the standard drug GCV (IC50 2.6–14.48 μM) (Efferth et al., 2002; Flobinus et al., 2014). Unexpectedly, DHA and artemisinin often showed minimal or undetectable activity. For instance, artemisinin showed significantly reduced activity (IC50 51.94 μM) compared to ART (IC50 4.37 μM) against AD169−GFP in human foreskin fibroblasts (HFFs), representing an approximately 10-fold difference (Chou et al., 2011). Similarly, DHA displayed an IC50 of 47.06 μM, much higher than that of ART (22.19 μM) in AD169-infected MRC-5 cells, whereas artemisinin showed no measurable activity in the same system (Flobinus et al., 2014). Lack of anti-CMV activity of artemisinin and DHA has been corroborated across several independent studies (Reiter et al., 2015; Çapcı Karagöz et al., 2018; Bock et al., 2017). The underlying molecular basis for these marked differences among structurally related monomers remains incompletely understood. One proposed explanation involves the unique side chain (OC(O)CH_2_CH_2_COOH) of ART, which may enhance its anti-CMV specificity (Chou et al., 2011). Additionally, the limited cellular uptake of artemisinin and DHA may contribute to their poor efficacy (Flobinus et al., 2014).

Novel artemisinin-derived dimers, trimers, and hybrid compounds exhibit a remarkable leap in anti-CMV efficacy. Representative derivatives such as dimer 838 (EC50 0.039 μM) (He et al., 2012), trimer TF27 (EC50 0.010–0.32 μM) (Hahn et al., 2020), and artemisinin–quinazoline hybrids with EC50 of 0.15 μM (Held et al., 2017), showing at least one order of magnitude increase in potency compared to artemisinin monomers.

#### 3.1.2 Structure-activity relationship

Anti−CMV activity of artemisinin derivatives is intrinsically linked to their structural features. The endoperoxide bridge, critical for artemisinin’s antimalarial mechanism, is also essential for anti−CMV activity, while derivatives lacking this bridge exhibit markedly reduced or absent activity. For instance, deoxyartemisinin, which lacks the endoperoxide, showed no interaction with the intermediate filament protein vimentin—a proposed CMV target (Roy et al., 2020). Among dimeric artemisinin derivatives, the diphenyl phosphate dimer compound 838 is highly active, whereas a deoxy dimer compound 574 lacking the endoperoxide bridge is inactive against CMV (He et al., 2011). Similarly, comparison of two related trioxane/sesquiterpene hybrids found that the endo (+) isomer showed strong anti−CMV activity with an IC50 of 0.67 μM, while the endo (−) isomer was inactive up to 10 μM, though the latter retained some mitochondrial effects (Wild et al., 2020a). In artemisinin−benzimidazole hybrids, 2−deoxy−artemisinin−based hybrids did not induce the mitochondrial structural conversion toward punctate patterns that were observed with active endoperoxide−containing compounds (Herrmann et al., 2023).

Beyond the endoperoxide bridge, other substituents in artemisinin derivatives also modulate activity. For example, in analogs of compound 832−4, the position and oxidation state of a sulfur atom influenced antiviral potency (He et al., 2011). Diphenyl phosphate-containing monomer and dimer artemisinin compounds display stronger inhibition and higher selectivity indices, and also by an irreversible mechanism, in contrast to the reversible inhibition seen with non-DPP derivatives (He et al., 2013). Overall, these results indicate that while the endoperoxide bridge is a primary determinant of anti−CMV activity, other moieties such as hemisuccinate side chains, DPP units, and sulfur-containing groups may also substantially influence the potency of artemisinin derivatives against CMV.

#### 3.1.3 Synergistic effects with conventional antiviral agent

In clinical CMV management, antiviral combination therapy like GCV with FOS represents a promising strategy to address viral drug resistance and reduce toxicity by enabling lower doses (El Chaer et al., 2016). Synergistic enhancement of viral inhibition has been observed between ART compounds and conventional anti-CMV agents, with no *in vitro* emergence of ART−resistant HCMV mutants reported, supporting the rationale for such combinations (Efferth et al., 2008; Roy et al., 2015). ART exhibits synergism with GCV, CDV, or MBV, and moderate synergism with FOS or LTV (Drouot et al., 2016). Similarly, the combination of GCV with the dimeric compound 838 or ART also displayed high synergy (He et al., 2012). Notably, several studies have reported synergistic antiviral effects between MBV and ART. In HFF cultures, adding 2 μM ART (below its IC50) reduced the IC50 of MBV against the wild−type strain T2211 from 12.1 μM to 0.20 μM (Chou et al., 2011). A synergistic effect between artemisone and MBV has been observed both *in vitro* and *ex vivo* placental models (Oiknine-Djian et al., 2019). Clinically, a triple combination therapy of ART, MBV, and donor-derived CMV-specific lymphocytes rapidly suppressed a drug-resistant CMV infection in an HSCT recipient (Stuehler et al., 2015). Since both MBV and ART primarily target drug-resistant CMV, their combination constitutes a viable treatment strategy that warrants further clinical evaluation.

#### 3.1.4 Dosing regimen optimization on ART’s activity

To compensate for the short half-life of artemisinin derivatives, they are clinically paired with longer-acting partner drugs such as lumefantrine or piperaquine in anti-malaria therapies to sustain efficacy and curb resistance (Eastman and Fidock, 2009; Li et al., 2022). Optimizing dosing schedules is also critical for antiviral activity. Time-of-addition experiments revealed that dividing the total ART dose into three or six fractional doses significantly lowered its IC50 from 4.31 ± 0.17 μM of a single dose to 2.34 ± 0.19 μM and 1.29 ± 0.08 μM, respectively. In contrast, this strategy did not alter the activity of GCV (Chou et al., 2011). A similar fractional-dosing–dependent enhancement was also observed for DHA (Flobinus et al., 2014).

#### 3.1.5 Influence of ferrous iron on ART’s activity

A ferrous iron (Fe²^+^)-dependent activation mechanism amplifies the bioactivity of artemisinin compounds. Fe^2+^ triggers the reductive cleavage of the endoperoxide bridge, generating carbon-centered radicals and reactive oxygen species (ROS) that drive parasiticidal actions (Haynes and Krishna, 2004). In the context of CMV infection, this Fe^2+^ mechanism underpins ART’s ability to counteract viral-induced adhesion molecule upregulation like Thy-1 (Kaptein et al., 2006). ART and DHA exhibit reduced anti-CMV activity in serum-starved cells with relative iron-depletion, corroborating the role of Fe²^+^ (Flobinus et al., 2014). Ferrocene-conjugated ART hybrids, which incorporate an iron source, exhibit superior potency (IC50 0.11–0.46 μM), further indicating that iron bioavailability may act as a critical adjuvant (Reiter et al., 2015).

### 3.2 *In vivo* study

According to the SYRCLE’s risk-of-bias tool, all four included *in vivo* studies showed complete outcome data without evidence of selective reporting. However, they provided insufficient detail on key methodological controls, including random sequence generation and comparability of baseline characteristics. Two of the four studies were additionally rated at high risk of other bias, primarily due to mismatched intervention protocols, such as differences in the route and frequency of administration between experimental groups.

HCMV has only one serotype and displays strict species specificity, infecting humans exclusively without cross-species transmission to animals. Despite this narrow host range, rodent cytomegaloviruses, particularly murine cytomegalovirus (MCMV), share substantial conservation with HCMV in core transcriptional regulatory mechanisms and functional protein domains (Fisher and Lloyd, 2020). This homology supports the use of well−established murine models as valuable tools in preclinical research (**Table 2**).

**Table 2:** *In vivo* studies on ART compounds against CMV.

Study	Animal model	Virus strain	Intervention	Outcome
(Kaptein et al., 2006)	Lewis rats, immunosuppressed by irradiation, 7 weeks	RCMV	ART (50 mg/kg p.o. qd, from -2 dpi to 15 dpi)	*In vitro*: Viral inhibition augmented with ferrous iron, reduced adhesion molecule Thy-1, and ART was synergistic with GCV, CDV, and FOS. *in vivo*: Reduced viral DNA load and virus titers in salivary glands.
(Wang et al., 2019)	BALB/c mice, 4–6 weeks	MCMV	ART (10 mg/kg p.o. bid, from 24 hpi, for 5 days), Synthetic ozonides of artemisinin OZ418 (10 mg/kg p.o. qd, from 24 hpi, for 5 days)	*In vitro*: OZ418 (EC50 9.8 ± 0.2 μM) was synergistic with GCV, inhibited cell cycle progression, reduced pRb and E2F1 expression, and downregulated CDKs expression. *in vivo*: T1/2 of OZ418 extended up to 3.4 h, once daily oral administration of OZ418 had significantly improved efficacy compared to twice daily oral ART.
(Sonntag et al., 2019)	C57BL/6j, Rag −/− mice, 6–8 weeks	MCMV	TF27 (5 mg/kg i.p. qd, from 1 dpi to 6 dpi)	*In vitro*: IC50 of TF 27 against MCMV was 0.06 ± 0.01 μM. *in vivo*: Restricted viral replication to the site of infection, 5 mg of TF27 produced similar efficacy as 30 mg of the positive control VGCV.
(Wild et al., 2020a)	BALB/c mice, 8 weeks	MCMV	TF27 (20 mg/kg p.o. qd) 3 kinds of dosing regimen: pre-infection (-2–0 dpi), therapeutic (0–3 dpi), and late-intervention (2–3 dpi)	*In vitro*: 2d pre-infection: TF27 (EC50 0.086 μM), while ART (EC50 >3 μM), LTV (EC50 0.532 μM). 7d post-infection: TF27 (EC50 0.040 μM), while ART (EC50 5.41 μM), LTV (EC50 0.003 μM). *in vivo*: TF27 significantly reduced spleen and liver viral load; antiviral efficacy was comparable to the positive control VGCV. TF27 exerted both prophylactic and therapeutic antiviral effects.

p.o., per os; i.p., intraperitoneal; qd, once daily; dpi, day post infection; hpi, hour post infection.

In an immunosuppressed rat model of RCMV infection, treatment of ART (50 mg/kg/day, from -2 to 15 dpi) combined with the iron supplement Ferrosanol (50mg/kg/day, from -7 to 15 dpi) significantly reduced viral DNA load in salivary glands (1.9×10³ vs. 1.5×10^4^ copies/μg tissue in untreated group) and decreased infectious titer by approximately 20-fold. This suggests that oral therapy with ART, possibly combined with ferrous iron supplement, may represent a potential therapeutic strategy for CMV infection (Kaptein et al., 2006).

The trimer derivative TF27 has shown notable efficacy. In immunodeficient Rag^−^/^−^ mice treated therapeutically (5 mg/kg/day, intraperitoneally) and immunocompetent BALB/c mice treated either prophylactically or therapeutically (20 mg/kg/day, orally), TF27 restricted viral dissemination to spleen and liver, effectively preventing organ dissemination. Its efficacy matched that of VGCV, and no adverse effects like weight loss were observed, indicating its potential utility in both prevention and treatment of CMV infection (Sonntag et al., 2019; Wild et al., 2020a).

### 3.3 Clinical data

This review included ten clinical studies evaluating the efficacy and safety of ART drugs for CMV infection: 3 cohort studies, 2 case series, and 5 case reports. Current evidence primarily comes from small-scale clinical reports, most of which involve combination therapy (**Table 3**). The independent antiviral efficacy of ART remains to be determined. Using the JBI critical appraisal tools, the overall quality of evidence was rated as low to moderate, limited by the absence of large randomized controlled trials (RCTs) focused specifically on CMV, heterogeneous dosing regimens, and inadequate adjustment for confounders in non−randomized studies.

**Table 3:** Clinical studies on ART drugs against CMV.

Study	Research type	Population	Intervention	Outcome
(Gantt et al., 2013)	A secondary cohort analysis from two randomized controlled trials	494 children with acute malaria	Artesunate-amodiaquine (containing ART 4 mg/kg/d p.o. for 3 days); comparison: sulfadoxine–pyrimethamine	CMV viremia was detected in 11.4% of children immediately before ART treatment and 10.7% 3 days later. No measurable difference in either the frequency or quantity of CMV detected in blood.
(Barger-Kamate et al., 2016)	Prospective cohort study	164 children with malaria and 143 non-malaria controls	Artemether-lumefantrine (containing artemether 20–40 mg/d p.o. for 14 days); comparison: no antimalaria treatment	Urine CMV viral loads were decreased in 164 children with malaria. In the subgroups of high-level CMV shedders, artemether-lumefantrine treatment was associated with a significant reduction in urinary CMV viral load.
(Gokarn et al., 2023)	Retrospective cohort study	25 HSCT patients (27 treatment episodes)	ART was mostly combined with GCV/FOS/leflunomide; monotherapy in 4 episodes. ART (adults: 120 mg bid; children: 2.4 mg/kg bid, maximum 120 mg, for 6–49 days)	ART achieved virological clearance in only 19% of treatment episodes, but limited replication of CMV in 74% of episodes at 2 weeks.
(Germi et al., 2014)	Case series	5 transplant recipients, including 2 HSCT and 3 solid-organ transplants	ART was combined with FOS/GCV. Case 1-3: ART (200 mg/d for within 1 month, 4 months, and 7 months, respectively); Case 4-5: ART (80 mg/d and 120 mg/d, respectively).	ART resulted in favorable virological and clinical responses in cases 1, 2, and 3 with mild CMV diseases (fever and neutropenia). By contrast, ART was ineffective in the other two cases with fatal CMV pneumonia, despite reductions in CMV DNA load in both blood and bronchoalveolar fluid.
(Wolf et al., 2011)	Case series	6 HSCT recipients	ART monotherapy (200 mg p.o. bid on day 1, followed by 100 mg qd, for 28 days)	Two patients showed a rapid log viral load reduction of 0.8–2.1 by day 7, with a viral decay half-life of 0.9–1.9 days. The other four patients showed a continued yet stalled viral growth slope during treatment. No adverse events were noted in treatment courses up to 28 days.
(Shapira et al., 2008)	Case report	a 12-year-old boy post-HSCT	ART monotherapy (100 mg/d p.o.)	The first 30-day treatment induced rapid viral load reduction within 10 days; a second course also led to rapid viral load decrease. A biphasic CMV viral decay curve was observed during the first treatment: an initial rapid decline phase(t1/2 = 0.9 days), followed by a slower decline phase (t1/2 = 8 days).
(Paccoud et al., 2022)	Case report	a 71-year-old female post-HSCT	ART was combined with immunomodulatory therapy of IL-2 and zoledronic acid. ART (120 mg/d initially, then 120 mg thrice weekly, for 7 months)	CMV viral load initially decreased by −0.8 log at day 7 and −1.3 log at day 14, but CMV infection relapsed after approximately 100 days of treatment.
(Muhamad et al., 2025)	Case report	a 7-month-old female infant post-HSCT	ART was combined with LTV, GCV, and leflunomide. ART (20 mg/kg/d)	Combination therapy successfully controlled a refractory, UL54-mutant CMV infection, enabling the discontinuation of all antiviral therapy at 3 months post-HSCT, with sustained virologic suppression

#### 3.3.1 Insights from malaria clinical studies

A secondary cohort analysis of two phase III RCTs evaluating ACTs for malaria was performed to indirectly assess their effects on CMV load (Gantt et al., 2013). Among 494 Ugandan children with malaria, a 3−day artesunate−amodiaquine regimen (containing 4 mg/kg ART) was compared with sulfadoxine−pyrimethamine. CMV was detected in 11.4% of children before treatment and 10.7% on day 3, with a mean viral load 0.30 log_10_ copies per million cells higher at day 3. No significant difference in CMV frequency or quantity was observed between treatment arms, likely because the short 3−day course was insufficient to exert a meaningful antiviral effect against CMV given its slow replication kinetics.

Another prospective cohort study compared 307 febrile children in Mali receiving ACTs (artemether−lumefantrine) for malaria with children with other illnesses not receiving anti-malaria treatment (Barger-Kamate et al., 2016). Viral loads were comparable on Day 0 and Day 3, but by Day 14 the median CMV load was lower in the ACTs group compared to the non-ACTs group (360 vs. 720 copies/mL, *P* = 0.059). Subgroup analysis indicated that ACTs led to a significant viral load reduction in children with high baseline viremia or CMV excretion in urine. Together, these findings suggest that artemisinin derivatives may require extended administration to suppress active CMV replication, with effects potentially more pronounced in individuals with higher viral burden.

#### 3.3.2 Cohort study in HSCT recipients​

A single-center retrospective cohort study evaluated 117 HSCT recipients with CMV reactivation (Gokarn et al., 2023). Among them, 25 patients (27 episodes) received ART after GCV failure or associated myelosuppression, at a dose of 120 mg twice daily for adults and 2.4 mg/kg twice daily (maximum 120 mg) for children. Complete viral clearance, defined as two consecutive negative PCR tests, was achieved in only 19% of the episodes. However, ART effectively prevented a 1-log increase of viral replication​ in 74% of cases within 2 weeks, suggesting a role in controlling viremia rather than achieving viral eradication.

#### 3.3.3 Case reports and case series in transplant recipients​

Early case reports suggested ART might serve as salvage therapy for drug-resistant CMV. The first case report described a 12-year-old HSCT recipient with GCV- and FOS-resistant CMV infection who received oral ART (100 mg/d) for 30 days. Viral load declined by 1.7–2.1-log within 10 days, with no rebound over 76 days, indicating ART’s potential to rapidly inhibit viral replication (Shapira et al., 2008). A case series of six HSCT recipients was treated preemptively with oral ART (200 mg twice on day 1, then 100 mg once daily for 28 days). Among them, two patients experienced a rapid viral load decline of 0.8–2.1-log within 7 days, with a viral decay half-life of 0.9–1.9 days, though four patients showed continued but stalled viral growth during treatment. Overall, ART’s antiviral response rates varied from 43% to 90%, apparently influenced by baseline viral growth dynamics (Wolf et al., 2011).

However, outcomes have been inconsistent. A renal transplant recipient with VGCV-resistant CMV colitis showing no significant virologic response to intravenous ART, at a dose of 180 mg/d, was later increased to 240 mg/day for 20 days, with clearance only achieved after switching to FOS (Lau et al., 2011). Similarly, in two cases of severe CMV pneumonia, ART reduced viral load in both blood and bronchoalveolar lavage fluid, yet both patients ultimately died of respiratory failure (Germi et al., 2014), highlighting the limited antiviral efficacy of ART in advanced CMV disease.

### 3.4 Mechanisms of ART against CMV

**Figure 5:**
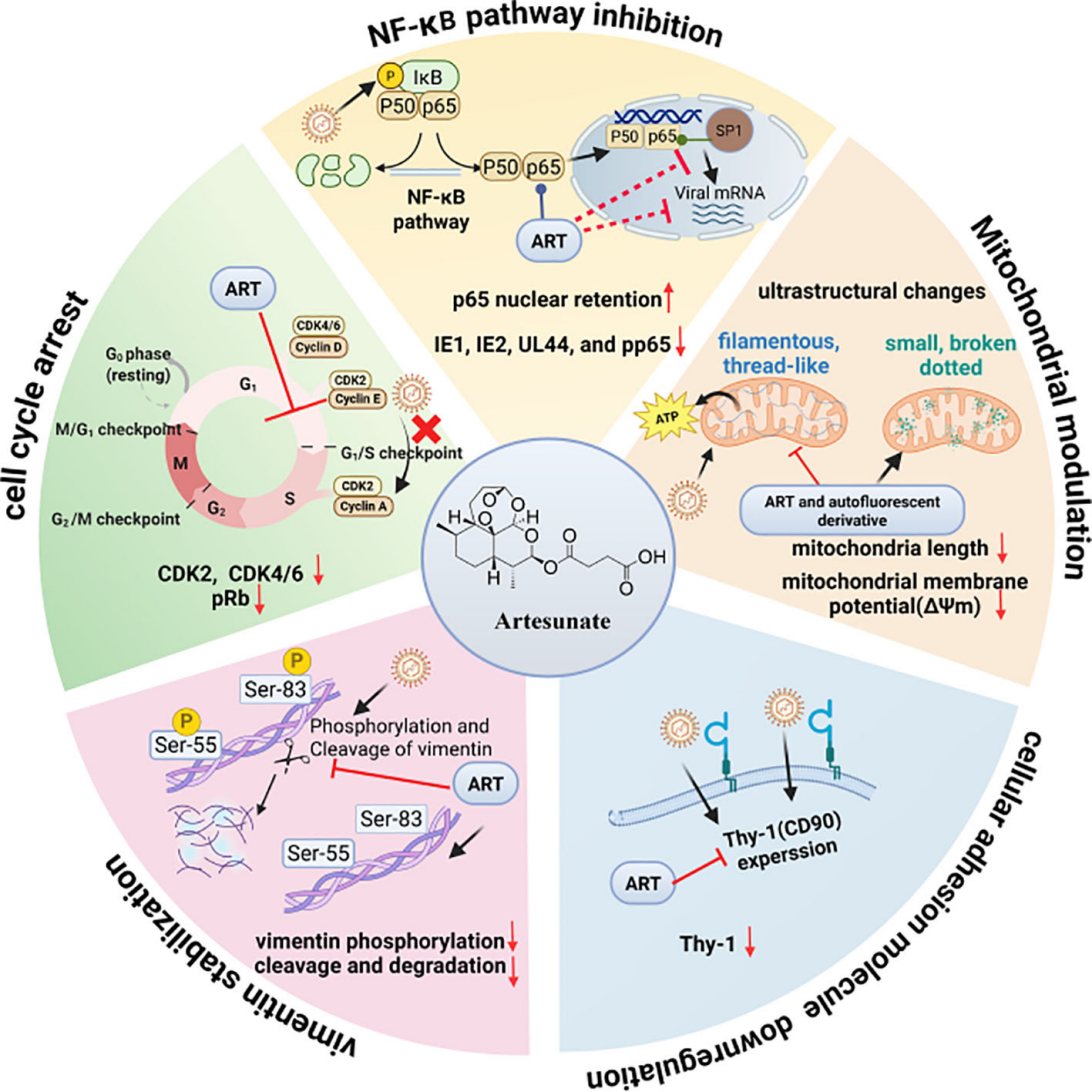
Potential mechanisms of ART against CMV. Created with BioRender.com.

Unlike conventional antiviral agents that directly target viral enzymes, current evidence predominantly supports a multimodal host-directed mechanism for ART, impacting several stages of the CMV replication cycle (**Figure 5**). This involves suppression of early-stage viral replication, along with additional effects after DNA synthesis. Nevertheless, the potential direct viral targets of ART have not been fully excluded, and further studies are needed to verify any distinct virus-directed effects.

#### 3.4.1 ART inhibits NF-κB pathway-mediated cell activation

The host NF-κB pathway mediates​ stage-specific effects during CMV infection. In the early phase, CMC rapidly induces NF-κB activation within minutes post-infection to promote viral replication by stimulating MIEP activity and IE gene expression via p65 nuclear translocation and cooperation with the transcription factor Sp1 (Compton et al., 2003; Sambucetti et al., 1989; Yurochko et al., 1995). During late infection, viral proteins such as UL26 block IκB degradation and NF-κB translocation to evade innate immune responses (Mathers et al., 2014).

ART reduces viral replication by selectively suppressing early NF-κB activation, typically by approximately 50%, without completely disrupting the pathway. This partial inhibition likely reflects the complexity of the NF-κB regulatory network, which is only partially accessible to drug-mediated suppression. Mechanistically, ART alkylates the p65 subunit, impairing its DNA-binding capacity and interaction with Sp1, as validated by immobilized ART pull-down assays and mass spectrometry analyses. ART treatment also suppresses NF-κB nuclear translocation by inducing p65 nuclear retention (46.0% vs. 5.3% in untreated cells) (Hutterer et al., 2015). These actions synergistically decrease the expression of viral IE1 and IE2 genes during the immediate-early stage, thereby suppressing subsequent viral replication steps (Efferth et al., 2002; Arav-Boger et al., 2010; He et al., 2013; Oiknine-Djian et al., 2018).

#### 3.4.2 ART enforces cell-cycle arrest

To enable replication of its large double-stranded DNA genomes, CMV triggers marked alterations in host cell cycle regulation—driving quiescent (G0) cells into G1 and inducing an unusual G1/S transition arrest (Spector, 2015). The virus sustains high cyclin-dependent kinases (CDK) activity to support its own replication in the E phase, while interfering with cellular DNA synthesis and division (Bogdanow et al., 2021). CDKs represent a positive regulator of CMV replication and a novel host-directed target for antiviral intervention (Obergfäll et al., 2024).

ART exerts potent anti−CMV activity via G0/G1 phase arrest of host cells. It effectively inhibits CMV replication in HFFs—whether contact-inhibited, serum-starved, or checkpoint-arrested at early/late G1. By contrast, ART was significantly less effective in actively cycling HFFs, where CMV replication was highly productive. Flow cytometry analysis confirmed ART blocks G1 to G1/S transition in CMV-infected HFFs, suggesting it promotes G0/G1 synchronization and disrupts the S-phase-like milieu required for efficient viral DNA synthesis. At the molecular level, ART downregulates key cell cycle regulators of CDKs (predominantly CDK2, plus CDK4/6) and suppresses retinoblastoma protein (pRb), indicating that its anti-CMV effect stems from cell cycle arrest (Roy et al., 2015). By disrupting these processes, ART primarily compromises the cellular conditions required for viral early (E) to late (L) gene expression and DNA replication.

#### 3.4.3 ART modulates mitochondrial function and morphology

As part of its survival strategy, CMV remodels host mitochondria both structurally and functionally. Driven by modulated mitochondrial biogenesis and fission/fusion dynamics, these changes help the virus to evade cell death, mitigate oxidative stress, and optimize bioenergetic and biosynthetic production (Monk and Zwezdaryk, 2020; Combs et al., 2020). Although ART is known to trigger mitochondrial dysfunction, ROS generation, and caspase activation to induce cytotoxic apoptosis in cancer cells (Zhang et al., 2018), its anti-CMV action follows a distinct pathway. ART and artemisinin−derived autofluorescent compound BG95 induced significant mitochondria shortening and reduced mitochondrial membrane potential at noncytotoxic concentrations (10μM of ART, 1μM of BG95), thereby inhibiting CMV replication without triggering apoptosis (Wild et al., 2020b; Hahn et al., 2020). Moreover, both BG95 and the trimeric compound TF27 could accumulate in mitochondria and elicit distinct ultrastructural changes—shifting mitochondria morphology from filamentous towards punctate (Herrmann et al., 2023).

#### 3.4.4 ART downregulates the cellular adhesion molecule

CMV upregulates host cell surface adhesion molecules, including ICAM-1 (CD54), TNFRSF5 (CD40), and Thy-1 (CD90), to facilitate cell-to-cell viral transmission (Martelius et al., 1998). Thy-1 modulation is linked to the viral glycoprotein complex gB/gH and host factors that couple viral entry to intracellular signaling cascades (Li et al., 2015).

ART significantly attenuated CMV-induced Thy-1 expression, in contrast to the limited efficacy observed with conventional antiviral agents. Quantitative flow cytometry analysis revealed that treatment with 15 μM of ART markedly reduced Thy-1 levels to 13.0% in infected GFP^+^ cells, compared to 29.8% in untreated infected cells, suggesting ART may exert immunoregulatory effects by specifically downregulating adhesion molecule Thy-1 (Kaptein et al., 2006).

#### 3.4.5 ART stabilizes vimentin

The host cytoskeleton plays a critical role in viral entry, replication, and egress (Kotsakis et al., 2001). CMV dynamically modulates the intermediate filament protein vimentin in a stage-specific manner. During early infection within 4 hpi, an intact vimentin network supports viral entry and intracellular trafficking (Miller and Hertel, 2009). As infection progresses (from 24 hpi onward), CMV induces vimentin phosphorylation at Ser55 and Ser83 and activates calpain, driving its disassembly and degradation to facilitate efficient viral dissemination. ART counteracts this process by reducing its phosphorylation and preventing its proteolytic cleavage, thereby stabilizing the vimentin network. Using an integrated approach, including mass spectrometry, co-immunoprecipitation, and surface plasmon resonance, vimentin was identified as a cellular target of ART. Binding relied on the bioactive endoperoxide bridge of ART, as the inactive analog deoxy-artemisinin showed no interaction. Antiviral activity of ART was markedly reduced (1.7-fold inhibition vs. 5.2-fold in controls) in vimentin knockdown fibroblasts, whereas the efficacy of GCV remained unaltered, supporting the functional relevance (Roy et al., 2020).

## 4 Conclusion and perspective

CMV infection continues to present a substantial clinical challenge, particularly in immunocompromised patients and neonates, especially those infected with drug-resistant strains or intolerant to conventional antivirals. Among clinically approved artemisinins, ART displays the most potent anti-CMV activity, exerting consistent inhibitory effects against both wild-type and drug-resistant CMV isolates *in vitro* with low micromolar IC50 values ranging from 1.30 to 22.19 μM, and shows preliminary efficacy in small-scale clinical reports. The endoperoxide bridge and hemisuccinate side chain represent key structural determinants of its antiviral potency. Novel optimized derivatives, including artemisinin-based dimers, trimers, and hybrid molecules, exhibit markedly enhanced activity with nanomolar IC50 values, though they remain at the preclinical stage.

Mechanistically, current evidence indicates that ART acts primarily through host-directed pathways essential for CMV replication, including suppression of NF-κB signaling, induction of G0/G1 cell-cycle arrest, modulation of mitochondrial dynamics, downregulation of adhesion molecules, and stabilization of vimentin. These distinctive mechanisms may theoretically reduce the risk of viral resistance and provide a rational basis for combination therapy with conventional anti-CMV agents. However, further validation is warranted during long-term clinical application.

Nevertheless, several critical obstacles hinder clinical translation. First, despite ART achieves high peak plasma concentrations (Cmax ~ 35.6 μM) after standard intravenous dosing, its extremely short half-life (typically minutes) results in only transient systemic exposure, which is likely insufficient to sustain antiviral pressure against slowly replicating CMV. These pharmacokinetic properties align with the variable clinical outcomes observed thus far, which are typically characterized by rapid viral load reduction rather than complete viral eradication. Moreover, the notable discrepancy between nanomolar antimalarial and micromolar anti-CMV potency of ART underscores an urgent need for rigorous pharmacokinetic–pharmacodynamic (PK/PD) studies to establish optimal dosing regimens specifically for antiviral therapy.

To fully realize ART’s therapeutic potential, future efforts may prioritize structural optimization and long-acting delivery systems, optimized dosing schedules to overcome its pharmacokinetic limitations. Alongside, well-designed randomized controlled trials are also warranted to validate its clinical efficacy and safety in high-risk patient populations. In summary, while ART represents a potential repurposing candidate for anti-CMV treatment, addressing its current translational gaps remains a considerable challenge.

## Data Availability

The original contributions presented in the study are included in the article/**Supplementary Material**. Further inquiries can be directed to the corresponding authors.

## Glossary

ART: artesunate
CMV: cytomegalovirus
HCMV: human cytomegalovirus
HHV-5: human herpesvirus 5
HIV: human immunodeficiency virus
MIEP: immediate−early promoter
IE: immediate early
E: early
L: late
GCV: ganciclovir
VGCV: valganciclovir
FOS: foscarnet
CDV: cidofovir
LTV: letermovir
MBV: maribavir
DHA: dihydroartemisinin
IV: intravenous
IM: intramuscular
Tmax: time to peak concentration
Cmax: peak concentration
T1/2: plasma half−life
WHO: World Health Organization
ACTs: artemisinin-based combination therapies
EBV: Epstein-Barr virus
HHV-6: human herpesvirus 6
IC50: half maximal inhibitory concentration
EC50: half maximal effective concentration
CC50: half maximal cytotoxic concentration
HELF: human embryonic lung fibroblasts
HFF: human foreskin fibroblasts
SI: selectivity index
MCMV: murine cytomegalovirus
PO: per oral
FCS: fetal calf serum
DPP: diphenyl phosphate
ROS: reactive oxygen species
NF-κB: nuclear factor kappa B
RCT: randomized controlled trial
CDK: cyclin-dependent kinases
pRb: retinoblastoma protein
