# Supplementary figures and images for: Artesunate repurposing for cytomegalovirus management: a systematic review of efficacy, mechanisms, and perspectives
Source: Front Cell Infect Microbiol. 2026 May 7;16:1815679. doi: 10.3389/fcimb.2026.1815679 (PMC13189954; doi:10.3389/fcimb.2026.1815679)

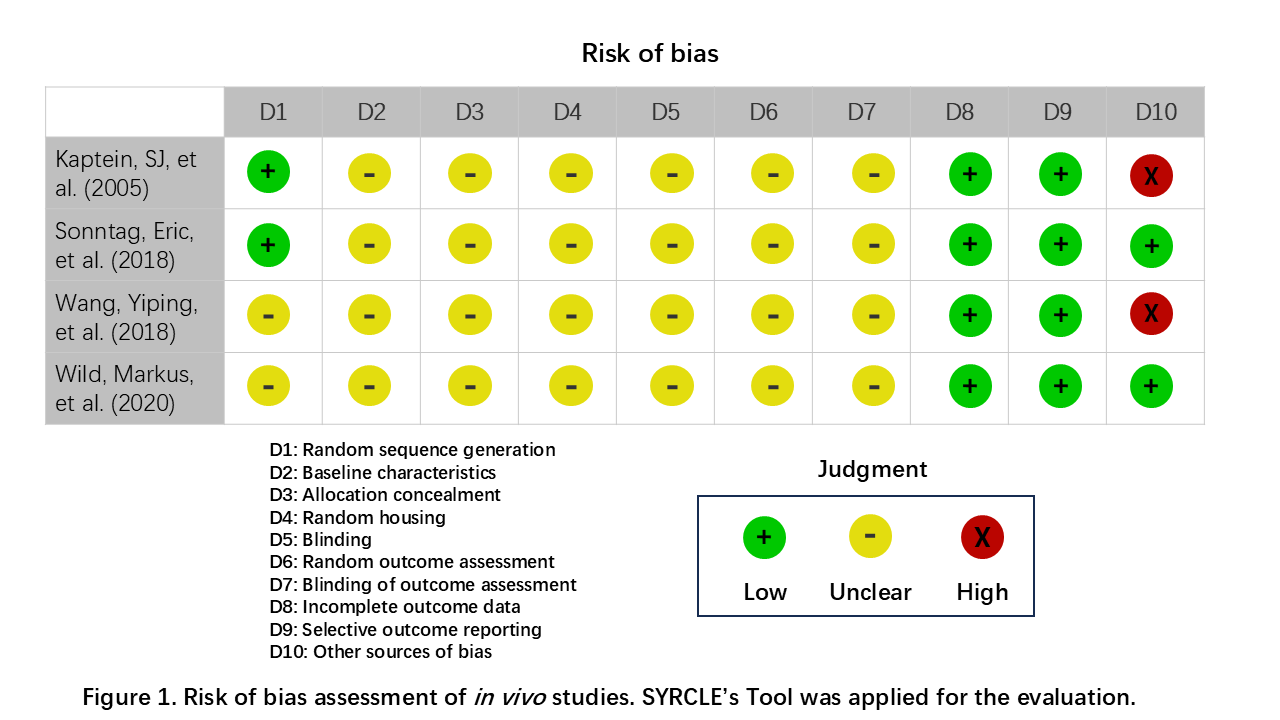

Supplement: Supplementary file 1 [file Image1.tif]

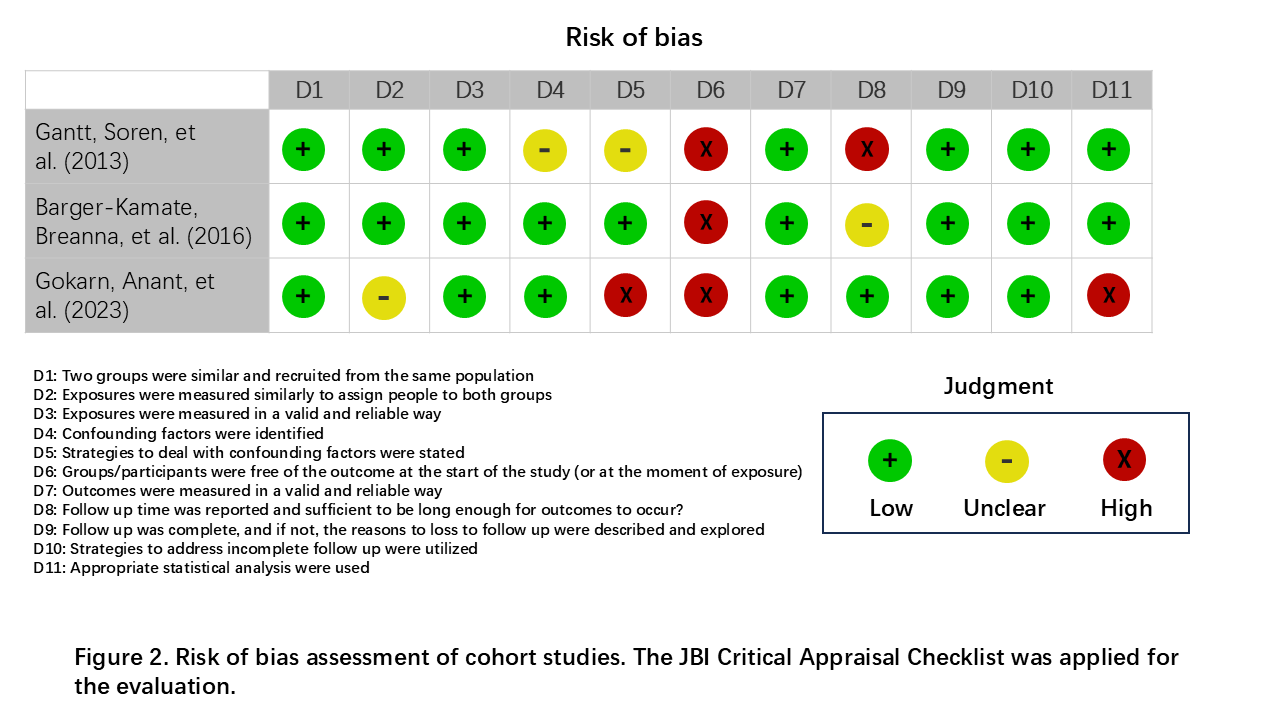

Supplement: Supplementary file 2 [file Image2.tif]

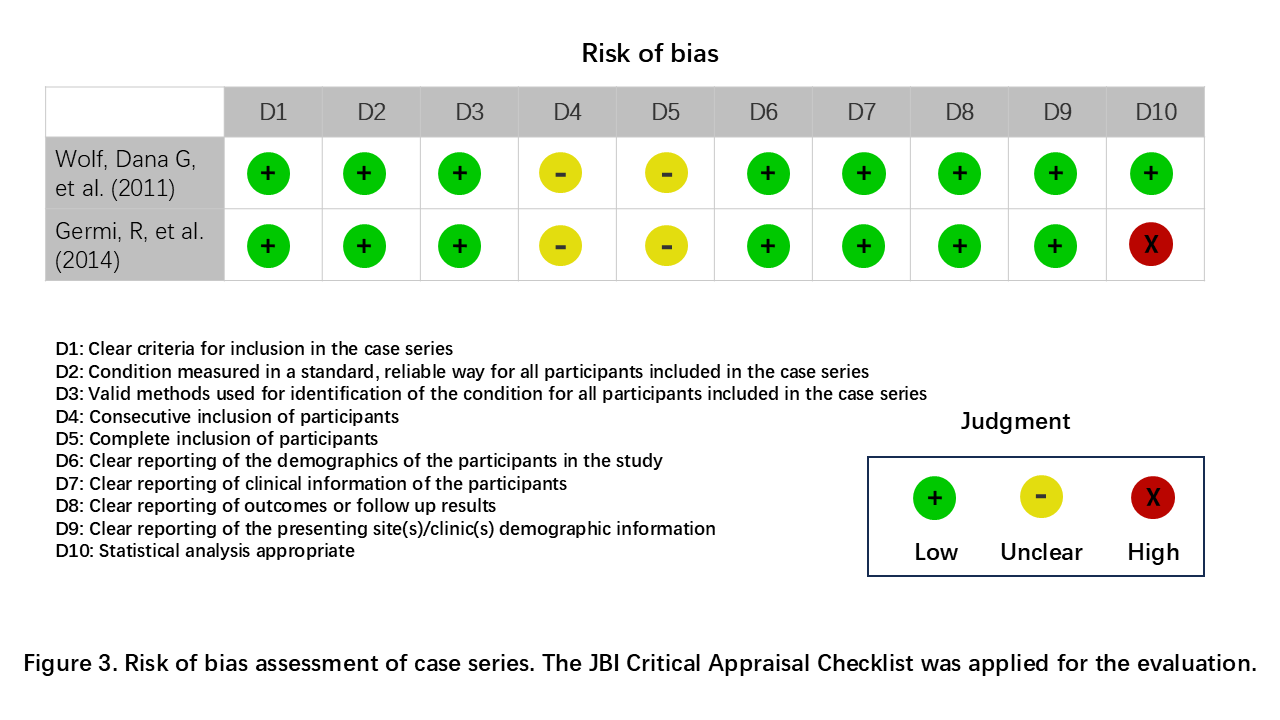

Supplement: Supplementary file 3 [file Image3.tif]

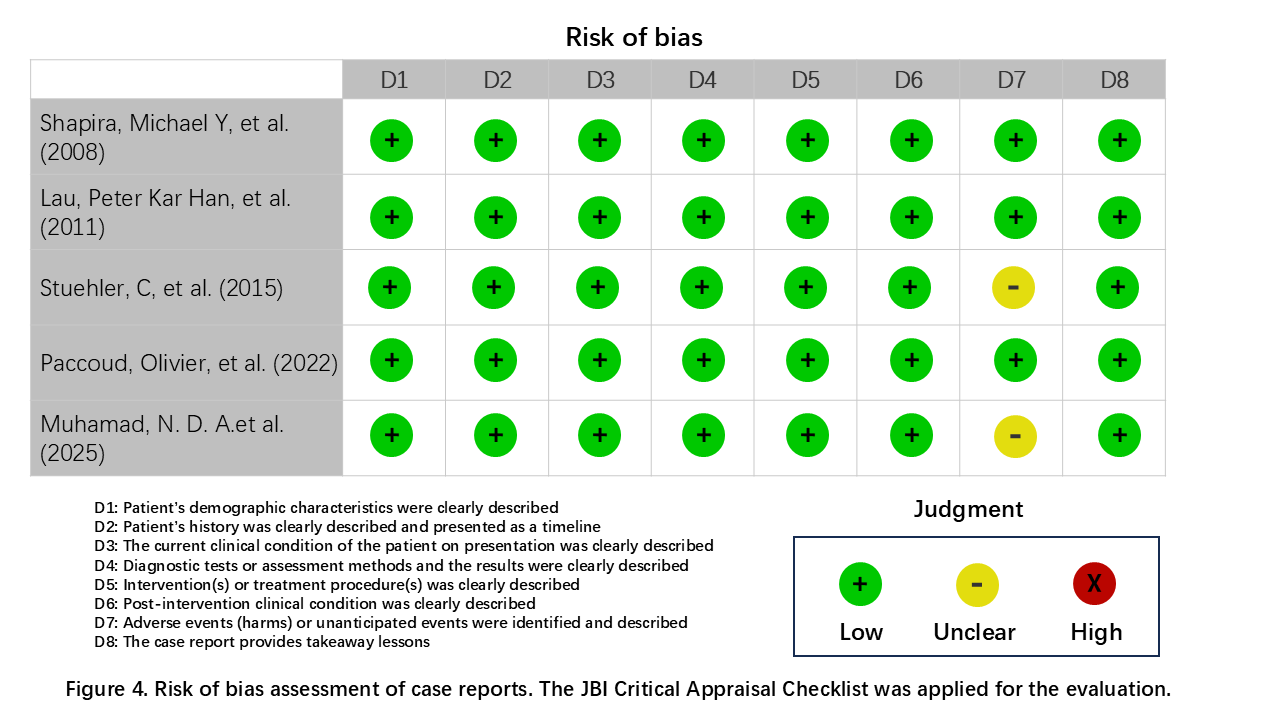

Supplement: Supplementary file 4 [file Image4.tif]
